# A polygenic risk score predicts mosaic loss of chromosome Y in circulating blood cells

**DOI:** 10.1186/s13578-021-00716-z

**Published:** 2021-12-12

**Authors:** Moeen Riaz, Jonas Mattisson, Galina Polekhina, Andrew Bakshi, Jonatan Halvardson, Marcus Danielsson, Adam Ameur, John McNeil, Lars A. Forsberg, Paul Lacaze

**Affiliations:** 1 Department of Epidemiology and Preventive Medicine, School of Public Health and Preventive Medicine, Monash University, Melbourne, Australia; 2 Department of Immunology, Genetics and Pathology, Science for Life Laboratory, Uppsala University, Uppsala, Sweden; 3 The Beijer Laboratory, Uppsala University, Uppsala, Sweden

**Keywords:** Mosaic loss of chromosome Y, LOY, mLOY, Polygenic risk score, PRS, ASPREE

## Abstract

**Background:**

Mosaic loss of Y chromosome (LOY) is the most common somatic change that occurs in circulating white blood cells of older men. LOY in leukocytes is associated with increased risk for all-cause mortality and a range of common disease such as hematological and non-hematological cancer, Alzheimer’s disease, and cardiovascular events. Recent genome-wide association studies identified up to 156 germline variants associated with risk of LOY. The objective of this study was to use these variants to calculate a novel polygenic risk score (PRS) for LOY, and to assess the predictive performance of this score in a large independent population of older men.

**Results:**

We calculated a PRS for LOY in 5131 men aged 70 years and older. Levels of LOY were estimated using microarrays and validated by whole genome sequencing. After adjusting for covariates, the PRS was a significant predictor of LOY (odds ratio [OR] = 1.74 per standard deviation of the PRS, 95% confidence intervals [CI] 1.62–1.86, p < 0.001). Men in the highest quintile of the PRS distribution had > fivefold higher risk of LOY than the lowest (OR = 5.05, 95% CI 4.05–6.32, p < 0.001). Adding the PRS to a LOY prediction model comprised of age, smoking and alcohol consumption significantly improved prediction (AUC = 0.628 [CI 0.61–0.64] to 0.695 [CI 0.67–0.71], p < 0.001).

**Conclusions:**

Our results suggest that a PRS for LOY could become a useful tool for risk prediction and targeted intervention for common disease in men.

**Supplementary Information:**

The online version contains supplementary material available at 10.1186/s13578-021-00716-z.

## Background

Mosaic loss of chromosome Y (LOY) refers to acquired Y-aneuploidy in a fraction of somatic cells. Population studies have identified LOY as the most common somatic change that occurs in circulating white blood cells of older men [1–10]. In serially studied men, the fraction of blood cells with LOY typically increases in frequency over time [2, 8–10]. For example, at least 40% of men aged 70 years in the UK Biobank were affected by LOY at baseline [5]. Single-cell analyses have identified that leukocytes with LOY are found in every studied older subject [11]. Epidemiological investigations show that the presence of LOY in blood leukocytes is associated with increased risk for all-cause mortality [2, 12] and a range of common diseases in men, such as hematological and non-hematological cancer [2, 10, 13–17], Alzheimer’s disease [3], autoimmune diseases [18, 19], cardiovascular events [12, 20], age-related macular degeneration [21] and type 2 diabetes [12]. The diverse range of associated outcomes suggest that LOY could act as a biomarker of generalized genomic instability [4, 5] as well as be linked with direct physiological effects; through impaired functions of affected leukocytes [2–6, 11, 17, 22–26]. Hence, identification of men with LOY occurring in peripheral blood could help to pinpoint men in the general population who are at the highest risk of common disease from an earlier age, for targeted intervention.

In addition to age, LOY is associated with smoking and air pollution, as well as other lifestyle factors [4, 9, 12, 27–29]. Furthermore, recent genome-wide association studies (GWAS) have identified up to 156 independent germline variants associated with risk of LOY occurring in leukocytes [4–6, 27, 29]. The LOY-associated germline risk variants are primarily enriched in genes related to DNA damage, cell-cycle regulation and cancer susceptibility [4, 5]. These variants can now be used to calculate a polygenic risk score (PRS) to predict individual propensity to be affected with LOY and thus, add genetic predisposition as a measurable risk factor for LOY beyond age and environmental exposures. The objective of this study was to calculate a novel PRS for LOY using previously the established germline risk variants (Additional file 1: Table S1) and to assess the predictive performance of this score in a large independent population of men aged 70 years and older. Our hypothesis was that a PRS for LOY could be used to improve risk prediction for LOY as men age, which in turn may help identify men with increased vulnerability for chronic and common disease, who could benefit from earlier targeted interventions.

## Results

### Baseline characteristics

The characteristics of the sample population are presented in Table 1. A total of 5131 DNA samples from males aged 70 years and older passed all QC metrics and were available for LOY analysis. The threshold for scoring of individuals with LOY was an mLRRY value based on array intensity data below − 0.06, representing LOY in at least 8.6% of the studied blood cells in a sample. Current smokers constituted a small percentage of the population (3.5%) and the majority of participants were current alcohol users (85.3%). The frequency of LOY among all participants was 27.2% based on the binary LOY threshold and we observed higher prevalence of LOY with age; affecting more than half of the participants aged 85 or older (Additional file 1: Table S2, Figures S1 and S2). Among the baseline characteristics, we found significant differences between men with and without LOY for age, smoking and alcohol use using the binary threshold (Table 1). No evidence of association between LOY and randomization to aspirin treatment was found.

**Table 1:** Characteristics of the sample population

	All (n = 5131)	LOY No (n = 3739)	LOY Yes (n = 1392)	*p*-value*
Age at Enrolment Mean (SD)	74.9 (4.18)	74.4 (3.84)	76.3 (4.73)	< 0.001
Smoking				< 0.001
Never	2253 (43.9%)	1680 (44.9%)	573 (41.2%)	
Former	2699 (52.6%)	1952 (52.2%)	747 (53.7%)	
Current	179 (3.5%)	107 (2.9%)	72 (5.2%)	
Alcohol				0.04
Never	461 (9.0%)	359 (9.6%)	102 (7.3%)	
Former	294 (5.7%)	211 (5.6%)	83 (6.0%)	
Current	4376 (85.3%)	3169 (84.8%)	1207 (86.7%)	
Treatment				0.57
Placebo	2564 (50.0%)	1878 (50.2%)	686 (49.3%)	
Aspirin	2567 (50.0%)	1861 (49.8%)	706 (50.7%)	
BMI (kg/m^2^)				
Normal	1063 (21.0%)	754 (20.5%)	309 (22.5%)	0.07
Underweight	38 (0.8%)	23 (0.6%)	15 (1.1%)	
Overweight	2633 (52.1%)	1921 (52.2%)	712 (51.9%)	
Obese	1298 (25.7%)	969 (26.3%)	329 (24.0%)	
Missing	18 (0.4%)	12 (0.3%)	6 (0.4%)	

^*^ Using the LOY binary variable, t-test or chi-square test we performed for continuous and categorical variables, respectively

### Comparison of PRS distribution in men with and without LOY

**Fig. 1:**
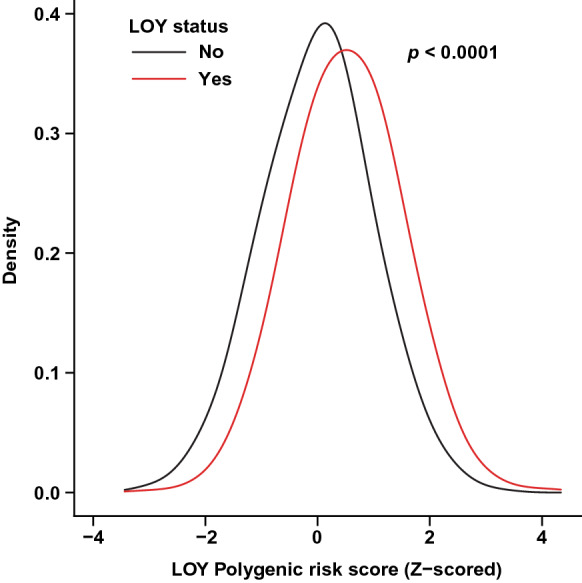
The distributions of polygenic risk scores for LOY (LOY-PRS) visualized by density plots among men with and without LOY. The *p*-value was calculated for the mean difference between the PRS distribution for participants with LOY (red) and without LOY (black) using ANCOVA, adjusted for age, smoking and alcohol use

We first sought to determine whether the overall PRS distribution in men with LOY had shifted compared to men without LOY. To investigate this, we plotted the PRS distributions side-by-side as density plots (Fig. 1) and tested for differences in the mean PRS distribution between the two groups, adjusted for age, smoking and alcohol use. We found that men with LOY displayed on average a higher PRS, as the mean distribution in men with LOY was shifted rightwards, versus men without LOY (ANCOVA, *p* < 0.001). This results thus validates a predictive performance of previously identified [5] risk variants in an independent cohort.

### Association of a Polygenic Risk Score with LOY mosaicism

Next, we tested for association between the LOY-PRS as a continuous variable and the binary LOY score. For each standard deviation increase in the PRS, we observed an odds ratio (OR) of 1.74 higher risk of LOY (95% confidence intervals [CI]  1.62–1.86, *p* < 0.001) after adjustment for age, smoking and alcohol use (Table 2). After this, we explored the LOY-PRS as a predictor of LOY risk in models adjusted for confounding effects of age, smoking and alcohol use. First, we investigated the predictive power of each risk factor independently, by comparing the area under the curve (AUC) in the separate models, in which LOY-PRS displayed the largest AUC (Additional file 1: Table S3). Then we compared the AUC of two LOY prediction models combining different risk factors; one including only age, smoking and alcohol use (AUC = 0.63, CI 0.61–0.65) and the second including also the LOY-PRS (AUC = 0.70, CI 0.68–0.71). Of note, a statistically significant improvement of the AUC was achieved by adding the LOY-PRS to the LOY risk prediction model (Additional file 1: Figure S3, *p* < 0.001).

**Table 2:** Association of a polygenic risk score for LOY predisposition (LOY-PRS) as a continuous variable, with LOY measured in 5131 men

	OR (95% CI)*	*p*-value*
LOY-PRS	1.74 (1.62; 1.86)	< 0.0001
Age, (years)	1.11 (1.09; 1.13)	< 0.0001
Smoking		
Never/former	Reference	
Current	2.13 (1.53; 2.94)	< 0.0001
Alcohol		
Never/former	Reference	
Current	1.21 (1.01; 1.47)	0.03

^*^ OR: odds ratios and *p*-values assessed using logistic regression

We then analysed the LOY-PRS as a categorical variable, comparing risk of LOY for participants in the lowest quintile of the PRS distribution (Q1, reference) versus those in the highest quintile of the distribution (Q5, high-risk group) and the middle 21–80% (Q2-4, middle group). We found that men in highest quintile of the PRS distribution had over fivefold higher risk of LOY than those in the lowest (OR = 5.05, CI 4.05–6.32, *p* < 0.001, Table 3). Similarly, compared with the lowest quintile, men in the middle 21–80% of the PRS distribution (middle group) also had a higher risk of LOY (OR = 2.23, CI 1.83–2.73, *p* < 0.001, Table 3), after adjusting for age, smoking and alcohol use. The increased risk of LOY observed for men in the high and middle PRS groups, compared with the low PRS group, was similarly observed when modelling LOY as a continuous variable (Additional file 1: Table S4).

**Table 3:** Association of a polygenic risk score for LOY predisposition (LOY-PRS) as a categorical variable (low, middle, high), with LOY measured in 5131 men

	OR (95% CI)*	*p*-value*
LOY low risk PRS	Reference	
LOY middle risk PRS	2.23 (1.83–2.73)	< 0.0001
LOY high risk PRS	5.05 (4.05; 6.32)	< 0.0001
Age, (years)	1.12 (1.09; 1.12)	< 0.0001
Smoking		
Never/Former	Reference	
Current	2.00 (1.44; 2.76)	< 0.0001
Alcohol		
Never/Former	Reference	
Current	1.2 (1.00; 1.45)	0.05

^*^ OR: odds ratios and *p-*values assessed using logistic regression. The LOY-PRS group define as: low < 20%, Middle 30–60% and high > 80% PRS

### Sub-group analysis by age

**Fig. 2:**
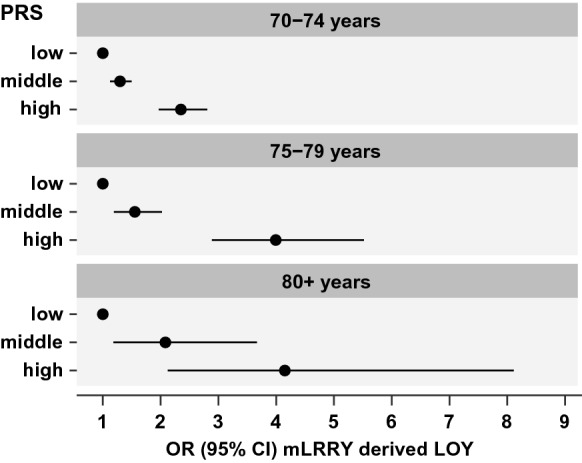
Association of the LOY-PRS with mLRRY-derived LOY increases with the age. The age dependence was evaluated by comparing results derived from the age groups 70–74, 75–79 and 80 + years, respectively. Within each age group, the predictive power of the PRS (estimated with odds ratios) is shown for men with low PRS (Q1 of PRS distribution; i.e. 0–20%), middle PRS (Q2-4; 21–80%) and high PRS (Q5; 81–100%)

To further investigate whether the PRS continued to be associated with higher risk of LOY as men age (e.g. independently of age), we stratified the cohort into three age-ranges; 70–74 years, 75–79 years and 80 + years and examined the effect of the PRS in each age group separately. These analyses showed that the association between the PRS and risk of LOY remained significant in each age range, and interestingly; that the strength of the PRS prediction increased with age (Fig. 2). Specifically, among participants aged 70–74 years, we observed an increased risk of LOY in the high PRS group (OR = 2.35, CI 1.97–2.81, *p* < 0.001) and in the middle group (OR = 1.30 CI 1.13–1.50, *p* < 0.001), versus the low group, after adjusting for smoking and alcohol use. Moreover, for men aged 75–79 years, we observed a stronger PRS effect than in the younger group, with a higher risk of LOY in the high PRS group (OR = 4.00, CI 2.90–5.52, *p* < 0.001) as well as the middle group (OR = 1.55, CI 1.19–2.02, *p* < 0.001). In the 80 + age-range, despite smaller participant numbers, we observed similar odds ratios compared with the 75–79 age-range, with higher risk of LOY in the high PRS group (OR = 4.14, CI 2.12–8.08, *p* < 0.001) and the middle group (OR = 2.09, CI 1.19–3.67, *p* < 0.010).

### Validation of LOY using whole genome sequencing data

The SNP array derived LOY estimation was validated using an orthogonal genomic technology. We performed a concordance analysis of LOY calls detected by microarray versus LOY calls based on whole genome sequencing (WGS) read depth, for a sub-set of 947 men for whom WGS data was available. The microarray-derived and WGS-derived LOY calls were highly correlated (Pearson correlation coefficient = 0.98) (Additional file 1: Figure S4).

## Discussion

Recent studies have provided insights into potential disease mechanisms that could help explain why men affected with LOY in blood cells live shorter lives. First, GWAS have identified germline variants associated with risk of LOY in leukocytes. Many of these risk variants are shared with loci for other diseases, and highlight genes involved in cell cycle regulation, DNA damage response and cancer susceptibility [4–6, 27, 29]. This ‘common soil’ of genetic predisposition helps, at least in part, to explain why men with LOY in peripheral blood display an increased risk for a range of different diseases, that may be mediated through age-related genomic instability in somatic tissues [5]. Second, it has been proposed that LOY in leukocytes could be linked with risk for disease in other organs by impaired immune functions of affected leukocytes [2, 3, 5, 7, 9, 22, 23, 25, 30]. This hypothesis is supported by studies suggesting involvement of chromosome Y in processes such as leukocyte development and function as well as transcriptional regulation [6, 11, 30–36]. For example, patients diagnosed with prostate cancer and Alzheimer’s disease might be affected with LOY in different types of immune cells, indicating a disease-specific link [11]. Furthermore, extreme down-regulation of chromosome Y genes (EDY) in different types of cancers [37] and in Alzheimer’s disease [38] demonstrates that expression of Y-linked genes could be important in the context of disease protection. Moreover, almost 500 autosomal genes have been shown to display LOY-associated transcriptional effect (LATE) by dysregulation in peripheral leukocytes with LOY, including many genes important for physiological immune functions [11]. Leukocytes with chromosome Y loss also display a reduced abundance of the cell surface immunoprotein CD99, encoded by a gene positioned in the pseudoautosomal regions of chromosomes X and Y, and essential for several key properties of leukocytes and immune system functions [26]. In aggregate, LOY in blood cells could either act as a barometer of genomic imbalance in- and outside of the hematopoietic system and furthermore, it is plausible that immune cells with this aneuploidy could be directly linked with disease etiology in human disease conditions with an immunological component.

In this study, we examined the predictive performance of a polygenic risk score (PRS) based on 156 previously-associated germline risk variants for LOY [5]. Using array data from 5131 healthy men aged 70 years and older, we found that the PRS was a significant predictor of LOY after adjusting for confounders, such as age, smoking and alcohol use. For each standard deviation increase in the PRS, we observed a 1.7-fold higher risk of LOY. Men in the highest quintile of the PRS distribution had, on average, more than fivefold higher risk of LOY compared with men in the lowest quintile of the distribution. A risk prediction model for LOY was improved significantly by the addition of the PRS to conventional risk factors such as age, smoking and alcohol use. Thus, regardless of the potential underlying mechanisms behind LOY associations with various disease outcomes discussed above, the results presented here show that the germline variation captured by the PRS can help identify men at highest risk of LOY in leukocytes. These results have implications for improved risk stratification and targeted intervention in ageing men.

We defined LOY using a microarray-derived signal intensity threshold, which corresponded to > 8.6% of cells losing the Y chromosome. We validated the microarray-derived LOY calls using WGS data. Based on the threshold, we found that the prevalence of LOY in the overall study population was 27.2%. After stratification by age, the frequency of men with LOY was 21%, 32%, 44% and 51% in men aged 70–74, 75–79, 80–84 and 85 years or older, respectively, consistent with previous reports [1–10]. Stratified analysis performed within age groups showed that the PRS was a significant predictor of LOY across all ages, with stronger predictive power in older men. This result fits well with previous data showing an accumulation of LOY with age, in the general population and an increased frequency of leukocytes with LOY in the blood of serially studied men [2, 8–10].

Strengths of our study include the well-characterized, older study population (mean age of 75 years at enrolment) with genotyping and WGS data available. A further strength is the ability of the ASPREE cohort to act as an independent validation of the germline variants identified from the UK Biobank population. Limitations of our study include the potential for survivorship bias in participant ascertainment, with individuals enrolled into the ASPREE study likely being healthier and at lower risk of disease than individuals from the general population in the same age range. Further, given that the majority of ASPREE participants were individuals of European genetic descent, this may limit the generalizability of our results to other ethnicities. We did not apply PRS refinement methods, such as effect size shrinkage or P-value thresholding, which could further improve PRS performance.

## Conclusions

Here we show that a PRS can be useful for identification of men with increased risk for LOY in leukocytes using a large population of older men. Mosaic LOY aneuploidy in leukocytes is associated with morbidity and mortality in populations of aging men, and constitutes a promising biomarker for general disease vulnerability. We report here that the inherited genetic make-up of individuals could be used to identify high-risk men with elevated likelihood of being affected with LOY during ageing, which could benefit early diagnosis and prevention of common disease. Implementation of a PRS for LOY risk prediction could promote earlier diagnoses of common disease, as well as enable risk stratification of men who would benefit more from early targeted intervention for a range of LOY-associated diseases.

## Methods

### Study population

This study was comprised of male participants of the ASPREE trial, a randomized, placebo-controlled trial investigating the effect of daily 100 mg aspirin on disability-free survival in healthy older individuals [39–41]. ASPREE inclusion criteria and baseline characteristics have been reported previously [42]. Briefly, individuals over the age of 70 years were enrolled, who had no previous history or current diagnosis of atherothrombotic cardiovascular disease events, dementia, loss of independence with basic activities of daily living, or any serious illness likely to cause death within five years, as confirmed by a general practitioner assessment. ASPREE participants also passed a global cognition screen at enrolment, scoring > 77 on the Modified Mini-Mental State (3MS) Examination. Participants were recruited 2010–2014 through general (family) practitioners in Australia and trial centres in the US.

### Microarray genotyping and imputation

We genotyped DNA from 6,140 peripheral blood samples provided by male participants at the time of study enrolment using the Axiom 2.0 Precision Medicine Diversity Research Array (PMDA) following standard protocols. To estimate population structure and ethnicity, we performed principal component analysis using the 1000 Genomes reference population (Additional file 1: Figure S5) [43]. Variant-level quality control included filters on > 90% genotyping rate and Hardy Weinberg-equilibrium, using plink version 1.9 [44]. Genotype data was imputed using the TOPMed server [45–47]. Post-imputation QC removed any variants with low imputation quality scores (r2 < 0.3).

### Estimation of LOY from microarray data

The level of LOY mosaicism in each participant was estimated using microarray intensity data from male-specific chromosome Y probes (MSY) as described in the Additional file and in Figures S6-S8. Briefly, Log R Ratio (LRR) output can be used to quantify copy number states from microarray data. The LRR is calculated as the logged ratio of the observed probe intensity to the expected intensity and observed LRR deviation in a specific genomic region is therefore indicative of copy number change. After quality control steps based on genotyping quality, sex, relatedness and ancestry; a total of 5131 male samples were retained for LOY analysis. For each sample, we first calculated the mLRRY as the median of the LRR values of the 488 Y-specific probes on the array, i.e. located within the MSY. The mLRRY is a continuous estimate of LOY; a value close to zero indicate a normal state while samples with LOY display mLRRY values below zero. To score samples with or without LOY we defined a threshold based on technical variation as described previously [9] and the percentage of cells with LOY in each participant was calculated [8]. We considered LOY as a continuous and categorical/binary variable in different analyses. Individuals with mLRRY less than -0.06 (equivalent to the 0.5th percentile of experimental error distribution) corresponding to > 8.6% of blood cells having LOY were considered as having LOY as a categorical variable.

### Estimation of LOY from whole genome sequencing data

We used whole genome sequencing (WGS) data that was available from 2795 ASPREE participants (male and female) through the Medical Genome Reference Bank project [48, 49]. WGS data was produced on the Illumina HiSeq X system with an average of 30 × sequencing coverage as described previously [49]. We compared microarray-derived and WGS-derived LRR calls using Pearson correlation in 947 male participants for whom both microarray and WGS data was available. LOY estimation from the WGS data was based on read depth, rather than LRR intensity differences. WGS data was analysed using the Control-FREEC software (version 11.5) [50] (details in Additional file 1).

### Calculation of polygenic risk score

The LOY polygenic risk score (LOY-PRS) was generated using 156 genome-wide significant variants previously associated with LOY [5]. A total of 123 variants passed genotyping and imputation QC thresholds and were present in the ASPREE imputed SNP array data set and were used to calculate the PRS (Additional file 1: Table S1). Plink version 2 was used to calculate the LOY-PRS as weighted sum of log odd ratios and effect alleles for each variant [51]. We categorized the LOY-PRS distribution into three groups based on quintiles (Q); low (Q1, 0–20%), middle (Q2-4, 21–80%) and high (Q5, 81–100%) risk.

### Statistical analysis

Baseline characteristics included age, smoking (current/former and never), alcohol use, body mass index (BMI) and treatment assignment (aspirin or placebo). Using the LOY binary variable, we performed a t-test or chi-square test for baseline continuous and categorical variables, respectively. We assessed the difference in LOY distribution by age using the Wilcoxon Test. The LOY-PRS distribution was Z-score standardised to have a mean 0 (SD 1) and tested for association in men with and without mLRRY-derived LOY using ANCOVA adjusting for age smoking and alcohol use. We than performed multivariable regression model for per standard deviation increase in LOY-PRS with mLRRY-derived LOY dichotomous and linear variable adjusting for baseline characteristics. In a separate regression model, the risk of mLRRY derived LOY (binary or continuous variable) was assessed between LOY-PRS categories using quintiles (Q) of the PRS distribution, considering the low-risk PRS group (Q1, 0–20%) as a reference, comparing against middle (Q2–4, 21–80% and high (Q5, 81–100%) risk groups. For sub-group analysis the LOY-PRS risk categories were further stratified into three age groups; 70–74 years, 75–79 years and 80 + years. Finally, the area under the curve (AUC) was calculated for age, smoking and alcohol use followed by adding LOY-PRS using receiver-operating-characteristics (ROC). We used DeLong’s test to compare the two ROC curves [52]. All analysis is performed using R version 4.0.3.

## Supplementary Information

**Additional file 1. **

## Data Availability

Genetic and phenotype data that support the findings of this study have been deposited in the European Genome-phenome Archive (EGAS00001005316 and EGAD00001005228).
